# Participation of Endothelin in the postnatal development of the rat kidney: Molecular mechanisms involved

**DOI:** 10.1371/journal.pone.0353524

**Published:** 2026-07-22

**Authors:** Lucas Humberto Oronel, María del Carmen Ortiz, Analía Czerniczyniec, Rocío Marinoni, María Ángeles Magnanelli, Silvia Lores Arnaiz, María Florencia Albertoni Borghese, Mónica P. Majowicz

**Affiliations:** 1 Facultad de Farmacia y Bioquímica, Departamento de Ciencias Biológicas, Cátedra de Biología Celular y Molecular, Universidad de Buenos Aires, Buenos Aires, Argentina; 2 Facultad de Farmacia y Bioquímica, Departamento de Ciencias Químicas, Cátedra de Fisicoquímica, Universidad de Buenos Aires, Buenos Aires, Argentina; 3 Consejo Nacional de Investigaciones Científicas y Técnicas (CONICET), Facultad de Farmacia y Bioquímica, Instituto de Bioquímica y Medicina Molecular Prof. Alberto Boveris (IBIMOL), Universidad de Buenos Aires, Buenos Aires, Argentina; 4 Consejo Nacional de Investigaciones Científicas y Técnicas (CONICET), Facultad de Farmacia y Bioquímica, Instituto de Química y fisicoquímica Biológicas (IQUIFIB), Universidad de Buenos Aires, Buenos Aires, Argentina; Royal College of Surgeons in Ireland, IRELAND

## Abstract

Some adult diseases such as hypertension and kidney disease may have their origins in early life, due to exposure to different adverse stressors. Previously we demonstrated that the administration of a dual endothelin receptor antagonist (ERA) to Sprague-Dawley (SD) rats from day 1–21 of life decreased glomerular number, predisposing adult male rats to salt sensitivity. This new study explores some early molecular mechanisms underlying the alterations observed in the kidneys of ERA-treated rats during the postnatal period, evaluating sex differences. Newborn male and female SD rats were treated with a dual ERA from day 1–6 and then sacrificed on day 7 of life to obtain the kidneys for the preparation of homogenates and mitochondrial fractions to assess: renal cell proliferation and apoptosis, nitric oxide synthases (Nos), neuronal isoform (Nos1) and endothelial isoform (Nos3) mRNA expression, NADPH- diaphorase (NADPH-d) activity, oxidative stress markers and antioxidant enzymes. ERA-treated male rats showed increased thiobarbituric acid-reacting substances (TBARS) and decreased nitric oxide (NO) to superoxide anion (O_2_^-^) ratio, with lower NADPH-d activity in the structures that give rise to glomeruli. Sex differences were observed in Nos1 and Nos3 mRNA expression, H_2_O_2_ production, and catalase activity, being females more protected than males. The alterations observed in the kidneys of ERA-treated rats during the early postnatal period could be due to a renal imbalance between NO and ROS, with increased oxidative stress, and a misbalance between proliferation and apoptosis. Our current findings show some molecular mechanisms underlying Endothelin inhibition in the early postnatal period, with potential utility for designing reprogramming strategies.

## Introduction

The kidney is vulnerable to early-life stressors. Thus, renal programming is key in the developmental programming of renal diseases. We have previously demonstrated that the inhibition of the endothelin (ET) system with a dual ET receptor antagonist (ERA) during the early postnatal period decreases the number of glomeruli, the juxtamedullary filtration surface area, and the glomerular filtration rate, while increasing proteinuria, being these effects more pronounced in male than in female rats [1]. Moreover, only ERA-treated male rats increased their blood pressure during adulthood after a high sodium diet, with an impaired ability to excrete sodium and water [2]. Considering the link between reduced glomerular number, hypertension and kidney disease programming [3,4], it is relevant to investigate the early molecular mechanisms that underlie those abnormalities in this experimental model.

Nephron development requires a complex interaction between growth factors, pro- and anti-apoptotic molecules, oxygen, and nutrients, all highly sensitive to perinatal disturbances, which can affect both its structure and function [5]. During development, apoptosis is essential for structural remodeling and to eliminate superfluous cells in a controlled manner [6], and its alterations can manifest as developmental abnormalities. Different studies have involved apoptosis as a determinant of nephron number [7]. Yoo et al. showed that the administration of an endothelin type A receptor (ETA) antagonist to neonatal rats decreases cellular proliferation and increases apoptosis in the kidney [8]. However, later studies in adult rats revealed that ETA triggers apoptosis in the kidney, while endothelin type B receptor (ETB) protects against it [9].

During kidney development, nitric oxide (NO) plays an important role in renal function regulation and apoptosis modulation [10,11]. Many reports support the role of oxidative stress in renal perinatal programming [12], proposing it as a link between perinatal adverse stressors and disease programming [13]. Moreover, oxidative stress is one of the main stimuli for the apoptotic intrinsic pathway [14].

Renal cells have a large mitochondrial density, enabling the kidney to sustain its increased metabolism [15]. Since mitochondria are also major sources of reactive oxygen species (ROS), when mitochondrial function is impaired, the kidney is particularly susceptible to oxidative stress [15]. Thus, a fine balance between NO and ROS is required to achieve normal nephrogenesis [16].

Additionally, although the severity of a negative developmental impact is often sex-dependent [17], the specific mechanisms causing these differences between males and females remain unclear [18]. Thus, we here hypothesized that the alterations observed in the kidneys of ERA-treated rats during the early postnatal period are due to an imbalance between NO and ROS, increasing renal oxidative stress. This leads to a misbalance between proliferation and apoptosis, decreasing cell proliferation and/or increasing apoptosis, especially in the structures that originate the glomeruli.

## Materials and methods

### Ethics statement

Protocols were complied with The ARRIVE guidelines 2.0 and were designed according to the Guide for Care and Use of Laboratory Animals from the National Institutes of Health, USA and the 9236/23 regulation of the Argentine National Drug Food and Medical Technology Administration (ANMAT), and approved by the Institutional Committee for Use and Care of Laboratory Animals (Comité Institucional para el uso y cuidado de Animales de Laboratorio; CICUAL) from Facultad de Farmacia y Bioquímica, Universidad de Buenos Aires (FFyB-UBA), Argentina (REDEC-2024–3207-E-UBA-DCT_FFYB). Efforts were made to use the minimum number of animals and minimize suffering.

### Animals and treatments

Male and female parent Sprague-Dawley (SD) rats (RRID: RGD_70508) were purchased from Facultad de Farmacia y Bioquímica, Universidad de Buenos Aires. Rats were housed in rooms with controlled temperature (22–24°C) and a 12-h dark-light cycle. Food and water were supplied *ad libitum*. After a week of acclimatization, adult female SD rats (70 days old weighing 230–250 g) were mated by exposure to a fertile SD male for 1 week. After birth, litter size was fixed at 10 ± 1. Newborn rats were treated daily from postnatal day (PD) 1–6 with vehicle or Bosentan (Actelion, 20 mg/kg/day; PubChem CID: 44387533), a dual ERA, administered orally with a micropipette. Four groups of animals were formed: control males (Cm), control females (Cf), ERA-treated males (ERAm), and ERA-treated females (ERAf).

On PD 7, the pups were sacrificed by decapitation, and the kidneys excised, weighed and processed or maintained at −80°C until future processing.

### Drugs and chemicals

The following drugs were purchased from Sigma Chemical Co.; St. Louis, Missouri, USA: sodium dodecyl sulfate (SDS), phosphotungstic acid hydrate (Cat No. 455970. PubChem Substance ID: 24869099), 1,1,3,3-tetramethoxypropane (MDA standard; Cat No. 108383), L-arginine, β-NADPH, dithiothreitol, SOD (Superoxide dismutase from bovine erythrocytes – lyophilized powder, ≥ 3000 units/mg protein (Cat No. S7571), Catalase, oxyhemoglobin (HbO2), Nx-nitro-L-arginine (L-NNA), N-nitro-L-arginine-methyl-ester (L-NAME), hydrogen peroxide (H2O2), epinephrine, mannitol, sucrose, NADH, succinate, rotenone, nitrobluetetrazolium, triton X-100, free-fatty acid bovine serum albumin (FFA-BSA), malate, glutamate, peroxidase from horseradish (HRP), reduced glutathione, glutathione reductase, tert-butyl hydroperoxide.

Origin of drugs and chemicals not named here are described in situ. Other general reagents or drugs used for preparation of buffers were of analytical grade.

### Tissue processing to prepare renal homogenates and mitochondrial fractions

*Homogenate preparation:* The kidneys were homogenized at 1076 g in MSTE buffer (230 mM mannitol, 70 mM sucrose, 10 mM Tris-HCl, 1 mM sodium EDTA, pH 7.4). Large tissue debris and nuclear fragments were removed by a low-speed spin (1000 g, 10 min, 4°C).

*Preparation of mitochondria-rich fraction and submitochondrial membranes:* The mitochondrial pellet was obtained by subjecting the supernatant to a second centrifugation step at 7650 g for 30 min, and then washed twice in MSTE buffer. Submitochondrial membranes were obtained by freezing and thawing the mitochondrial preparation twice and then homogenized by passage through a 27G needle. This preparation consisted of a fraction of outer and inner membranes, without restrictions to substrate access [19].

Protein and phosphatase inhibitors (Calbiochem, Merck; Darmstadt, Germany) were added to all the samples.

Protein concentration was measured using a BCA^TM^ Protein Assay Kit (Pierce, Rockford, IL, USA). Absorbance was read using an Infinite F50 microplate reader (Tecan Trader AG, Switzerland) at 560 nm.

### Cellular proliferation evaluation

Cellular proliferation was evaluated by immunohistochemistry using a primary rabbit polyclonal antibody specific for Proliferating Cell Nuclear Antigen (PCNA) (Rabbit polyclonal anti-PCNA antibody (ab18197); AbCaM), Cambridge, UK.), followed by a polyclonal secondary biotinylated goat anti-rabbit (Goat anti-Rabbit IgG (H + L) Secondary Antibody, Biotin. Catalog # 65–6140; Invitrogen / Thermo Fischer; Waltham, Massachusetts, USA). Positive cells were detected with an avidin-biotin-based peroxidase detection kit using 3,3’-diaminobenzidine as the chromogen (Vectastain ABC kit, (Cat No. PK6100), Vector Laboratories, Burlingame, CA, USA). Tissue sections were counterstained with hematoxylin (Cod. 9491.08; Biopack, Argentina). Negative controls were performed by omitting the primary antibody, and endogenous peroxidase activity was quenched with hydrogen peroxide to prevent unspecific staining.

The number of PCNA-positive nuclei present in the immature structures that originate the glomeruli was counted in ten cortical and ten juxtamedullary fields from four animals per group (total magnification 400x) and representative images were taken using an Olympus 8J15816 light microscope coupled to a digital camera (Qcolor 3, Olympus America, Inc., Richmond Hill, Ontario, Canada). All determinations were performed blindly and under standardized conditions by the same researcher.

### Apoptosis evaluation

Apoptosis was evaluated using the Terminal deoxynucleotidyl transferase (TdT) dUTP Nick-End Labeling (TUNEL) assay. The *in Situ* Cell Death Detection Kit, AP (Roche Diagnostics GmbH, Mannheim, Germany, Ref 11 684 809 910), a non-radioactive technique designed to end-label the fragmented DNA of apoptotic cells, was used. Renal tissue sections of 4 μm were deparaffinized in xylene and rehydrated in graded ethanol and phosphate buffer saline (PBS). Then, the sections were incubated in proteinase K solution (20 μg/mL in PBS), (Merck, Darmstadt, Germany) for permeabilization. Tissue slides were treated with the fluorescein d-UTP-labeled nucleotide mix and the terminal TdT enzyme (TUNEL reaction mixture) in the reaction buffer for 60 min at 37 °C to allow the end-labeling reaction. The incorporated fluorescein was detected by anti-fluorescein antibody provided in the kit, conjugated with alkaline phosphatase (converter AP). The sections were stained with BCIP/NBT color development substrate (Promega, Madison, WI, USA), counterstained with hematoxylin and mounted in PBS/glycerol for light microscopy. The number of TUNEL-positive nuclei was counted in ten cortical and ten juxtamedullary fields from four animals of each group (total magnification 400x) and representative images were captured using a Nikon Model Eclipse E200 light microscope (Nikon Instrument Group, Melville, NY, USA). All determinations were performed blindly and under standardized conditions by the same researcher.

### Evaluation of TBARS production

Thiobarbituric acid-reacting substances (TBARS) are commonly used markers for lipid peroxidation and malondialdehyde (MDA) production. The amount of TBARS production was determined by a fluorescence assay in renal homogenates.

Homogenates were treated with 1 mL of 0.1 N sodium dodecyl sulfate, 0.1 N HCl, 0.15 mL of 10% phosphotungstic acid and 0.5 mL of 0.7% 2-thiobarbituric acid (Merck, Darmstadt, Germany). After 1 h of boiling, the samples were cooled, and 2.5 mL of butanol was added. Fluorescence of the butanol layer was measured at 515 nm for excitation and 555 nm for emission. The values were expressed as nmoles of MDA per mg of protein, using a MDA standard prepared from 1,1,3,3-tetramethoxypropane [19].

### Evaluation of nitric oxide production

NO production was measured in submitochondrial membranes by using a spectrophotometric method, following the oxidation of oxyhemoglobin (HbO_2_) to methemoglobin (metHb) at 37 °C. This assay was performed using a Beckman Coulter Series DU 7400 diode array spectrophotometer in which the active wavelength was set at 577 nm and the reference wavelength at the isosbestic point at 591 nm (e = 11.2 mM/cm). The reaction medium contained: 50 mM phosphate buffer pH 5.8, 1 mM CaCl2, 50 μM L-arginine, 100 μM β-NADPH, 10 μM dithiothreitol, 4 μM Cu–Zn superoxide dismutase (SOD), 0.1 μM catalase, 0.5–1.0 mg submitochondrial protein/mL, and 25 μM HbO_2_ (expressed per heme group). HbO_2_ reacts efficiently with NO if the reaction between NO and O_2_^–^ is prevented. SOD is added to abrogate any other reaction with O_2_^-^, including a direct oxidation of HbO_2_ to metHb or reduction of metHb to hemoglobin. Since in the presence of SOD, H_2_O_2_ could be produced and consequently oxidize both HbO_2_ and metHb, catalase was added.Controls adding 0.5 mM Nx-nitro-L-arginine (L-NNA) and 0.5 mM N-nitro-L-arginine-methyl-ester (L-NAME) as NOS inhibitors were performed in all cases to give specificity to the assay; addition of L-NNA and L-NAME inhibited the rate of hemoglobin oxidation by about 73% and 50% respectively [19,20].

### Evaluation of superoxide anion (O_2_-) production

The rate of O_2_^-^ production was evaluated in submitochondrial membranes, measuring the O_2_^-^-dependent oxidation of epinephrine to adrenochrome at 37°C (ε_480 nm_ = 4.0 mM^-1^ cm^-1^) [21]. The reaction medium contained: 1 mM epinephrine, 0.23 M mannitol, 0.07 M sucrose, 0.1 μM catalase, 20 mM Tris-HCl pH 7.4, and submitochondrial particles (0.15 mg protein/mL). A 60 mM epinephrine bitartrate solution, pH 2.0, kept in ice, was diluted in the reaction medium immediately before use. SOD was used at 0.1–0.3 µM final concentration to give assay specificity [22].

Submitochondrial membranes were supplemented with substrates and inhibitors to obtain maximal O_2_^-^ production. NADH (50 μM) and succinate (7 mM) were used as substrates and rotenone (1 µM) as a specific inhibitor to evaluate O_2_^-^ production at the NADH dehydrogenase and at the ubiquinone-cytochrome b region, respectively.

### Determination of Nos1 and Nos3 mRNA expression

To determine nitric oxide synthases (NOS) *Nos1* (neuronal isoform) and *Nos3* (endothelial isoform) mRNA expression, total RNA was isolated from renal homogenates using the SV total RNA Isolation System (Promega, Madison, WI, USA). Total RNA was reverse-transcribed to cDNA using a high-capacity reverse transcription kit (A&B – Applied Biosystems, CA, USA). For real-time detection of *Nos1* and *Nos3* transcripts and the reference gene (Gapdh), MezclaReal (Real-Time PCR commercial mixture from Biodynamics, Argentina) and specific primers (For *Nos1* and *Nos3*: IDT -Integrated DNA Technologies and for *Gapdh*: GBT Oligos) were used (Table 1). The normalized gene expression method (2^–∆∆CT^) for the relative quantification of gene expression was used. The difference in the cycle threshold (CT) for *Nos1*/*Nos3* and *Gapdh* for control rats was subtracted from the difference in the CT for *Nos1*/*Nos3* and *Gapdh* for each of the experimental groups [23]. The following formula was applied: ∆∆CT = (CT_*NOS*_ – CT_*GAPDH*_) experimental – (CT_*NOS*_ – CT_*GAPDH*_) control rats).

**Table 1:** Sequences of the Real-Time PCR primers used.

Target gene	Forward primer (5´-3´)	Reverse primer (5´-3´)
*Gapdh*	GAAGGGCTCATGACCACAGT	GGATGCAGGGATGATGTTCT
*Nos1*	CTGCAAAGCCCTAAGTCCAG	AGTGTTCCTCTCCTCCAGCA
*Nos3*	GCAAGACCGATTACACGACA	GCTCTCAGGAGGTCTTGCAC

The real-time PCR started at 94°C for 2 min and was followed by 35 thermal cycles at 94°C for 15 s, 58°C for 35 s and 72°C for 30 s.

### Determination of NADPH-diaphorase activity in cortical renal structures

To determine NADPH-diaphorase (NADPH-d) activity, renal tissue was processed using the NADPH-d histochemical method according to Rothe et al. [24]. This method can detect cells containing any of the NOS isoforms [25], and can be used to monitor NOS activity at a cellular level. The kidneys were fixed with 4% paraformaldehyde in 0.1 M phosphate buffer pH 7.4, cryoprotected with sucrose, frozen, sectioned at 12 µm, and mounted on gelatin-coated glass slides. Sections were simultaneously incubated at 37°C for 1 h in a reaction mixture containing 0.1% β-NADPH and 0.02% nitrobluetetrazolium diluted in 0.1 M phosphate buffer with 0.3% Triton X-100 and then mounted in PBS/glycerol (1:3). Observation, optical density (OD) measurement, and photography were made with a Nikon Alphaphot-2 YS2 coupled to a SONY camera Model No. SSC-DC50A (magnification 400x). NADPH-d-stained cells were measured by Image J. Each set of OD measurements (control and experimental groups) was performed blindly and under standardized conditions. After automatic normalization of grayscale, the same interactive delineation and contrast enhancement of all images was performed. This process was done in different renal structures of the same section and different sections of the same kidney [26].

### Determination of the hydrogen peroxide (H_2_O_2_) production rate

The hydrogen peroxide (H_2_O_2_) production rate was determined in freshly isolated mitochondria (0.2 μg protein/μL) by the Amplex Red-horseradish peroxidase (HRP) fluorescent assay (λ_EXCITATION_ = 563 nm/λ_EMISSION_ = 587 nm) using a Varioskan® LUX microplate reader at 30 °C. Buffer solution (125 mM sucrose, 65 mM KCl, 10 mM HEPES, 2 mM KH_2_PO_4_, 1 M MgCl_2_, 0.01% PFA-BSA; pH 7.2), 2 mM malate, and 5 mM glutamate were supplemented with 25 μM Amplex™ Red (Invitrogen /Thermo Fischer; Waltham, Massachusetts, USA; Cat No. A36006) and 0.5 U mL^-1^ HRP, and samples were added.

A H_2_O_2_ standard solution was used for calibration (ε_240 nm_ = 43.6 M^-1^ cm^-1^), and results were expressed as nmol H_2_O_2_/min.mg protein [27].

### Determination of catalase activity

Catalase activity was determined in submitochondrial membranes by spectrophotometry, following absorbance decrease of H_2_O_2_ at 240 nm, in a medium containing 50 mM phosphate buffer and 10 mM H_2_0_2_ (pH 7.2), and expressed as pmoles enzyme/min mg protein [28].

### Determination of glutathione peroxidase activity

Glutathione peroxidase (GPx) activity was measured in submitochondrial membranes following NADPH oxidation at 340 nm (ε_340 nm_ = 6.22 mM^-1^ cm^-1^) in the presence of 0.17 mM reduced glutathione, 0.2 U/mL glutathione reductase and 0.5 mM tert-butyl hydroperoxide and expressed as µmol/min mg protein [28].

### Statistical analysis

Data were analyzed using two-way ANOVA, with treatment and sex as factors. The main effect of each factor and the interaction between the factors were tested. Bonferroni´s post-test was used for multiple comparisons. Data are presented as the mean ± standard error of the mean. GraphPad Prism version 8.0 project for Windows, GraphPad Software (San Diego, CA, USA) was used. The null hypothesis was rejected when p < 0.05.

Non-continuous variables such as the number of PCNA-positive nuclei and the number of TUNEL-positive nuclei were analyzed using the non-parametric Scheirer-Ray-Hare test. The main effect of each factor and the interaction between both factors were tested. The non-parametric post-hoc test Mann–Whitney U (Wilcoxon rank-sum) for multiple comparisons was used. These data were analyzed using R 3.0.2 for Windows and expressed as the median and the minimum-maximum range. A significant difference was considered when p < 0.05.

## Results

To investigate the molecular basis of the previously reported glomeruli defect in postnatally ERA-treated rats (1), we measured body and renal weights, markers of cell proliferation and apoptosis, nitric oxide signaling, oxidative stress and antioxidant defenses in control and ERA-treated 7-day-old rats.

### Animal body and renal weights

No significant differences were observed in body weight between experimental groups on PD 7. Similarly, no significant differences were observed in kidney weight or kidney weight/100 g b.w. between experimental groups. Results are shown in Table 2.

**Table 2:** Growth parameters in control and ERA-treated 7-day-old rats.

	Cm (n = 16)	ERAm (n = 19)	Cf (n = 19)	ERAf (n = 19)
Body weight (g)	17.50 ± 0.53	17.39 ± 0.41	18.00 ± 0.48	17.95 ± 0.55
Renal weight (g)	0.098 ± 0.005	0.094 ± 0. 003	0.103 ± 0.004	0.101 ± 0.004
Renal weight (g)/100 g b.w.	0.552 ± 0.014	0.542 ± 0.009	0.567 ± 0. 010	0.564 ± 0. 010

Values are mean ± SEM. Data were analyzed using two-way ANOVA followed by the Bonferroni post-test. ERA treatment and sex had no significant effect on any of the parameters tested. Cm: control males; ERAm: ERA-treated males; Cf: control females; ERAf: ERA-treated females.

### Cellular proliferation and apoptosis

**Fig 1:**
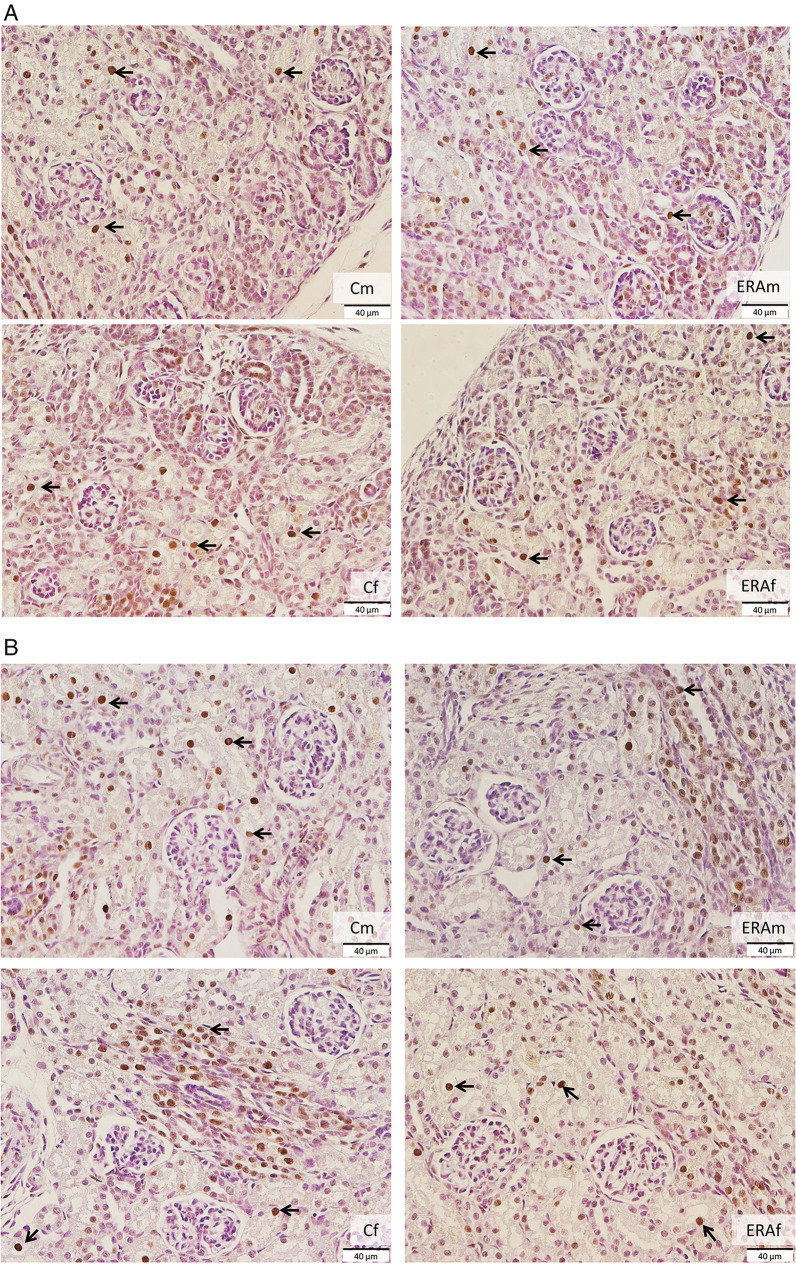
Cellular proliferation assessment by PCNA immunohistochemistry in the renal cortex of 7-day-old rats. 1A: Representative images of PCNA immunohistochemistry in the cortical area; **1B:** Representative images of PCNA immunohistochemistry in the juxtamedullary area. Cm: control males; ERAm: ERA-treated males; Cf: control females; ERAf: ERA-treated females. Black arrows show some positive nuclei. Total magnification: 400x.

**Fig 2:**
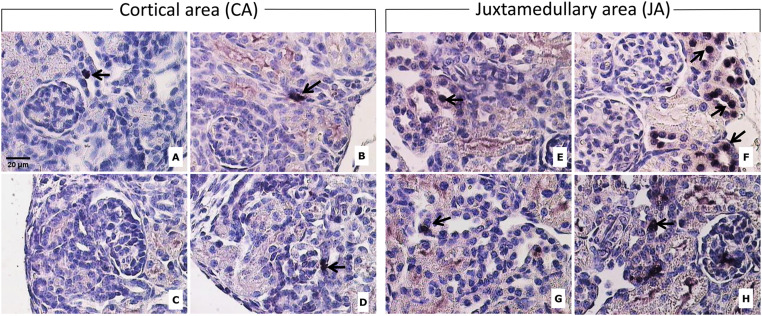
Cellular death assessment by TUNEL assay in the renal cortex of 7-day-old rats. Representative images for TUNEL assay in the renal cortex; CA: cortical area; JA: juxtamedullary area. A = control males (CA); B = ERA-treated males (CA); C = control females (CA); D = ERA-treated females (CA). E = control males (JA); F = ERA-treated males (JA); G = control females (JA); H = ERA-treated females (JA). Black arrows show some positive nuclei. Total magnification: 400x.

ERA treatment did not modify renal cellular proliferation (evaluated by PCNA expression) in the cortical area or in the juxtamedullary area. On the other hand, cellular death (assessed by TUNEL assay) showed a significant interaction between sex and ERA treatment in the juxtamedullary area (p < 0.001). Both male and female ERA-treated rats showed a higher number of apoptotic nuclei than their respective controls. However, while ERAm increased the number of apoptotic nuclei 8-fold, ERAf increased it only 4-fold versus their respective control. These results are shown in Table 3 and representative images in Figs 1A, 1B, and 2.

**Table 3:** Cellular proliferation and apoptosis evaluated by PCNA and TUNEL in the renal cortex of 7-day old rats.

	Cm	ERAm	Cf	ERAf
PCNA (CA)	368.5 (317-639)	392.5 (229-610)	523.5 (380-930)	482 (174-943)
PCNA (JA)	663 (623-773)	679.5 (546-697)	871 (805-877)	617.5 (407-914)
TUNEL (CA)	8.5 (5-16)	13.5 (8-17)	11.5 (9-15)	17.5 (8-22)
TUNEL (JA)	11.5 (7-26)	95* (25-113)	7.0 (6-11)	33.5**#** (14-82)

PCNA: Proliferating Cell Nuclear Antigen; TUNEL: Terminal deoxynucleotidyl transferase (TdT) dUTP Nick-End Labeling assay. The number of positive nuclei per 10 fields was counted for each experimental unit. Results are expressed as the median, representing the central value, along with the minimum and maximum values, indicating the range of the data (PCNA: n = 4/experimental group; TUNEL: n = 4/experimental group). * p < 0.05 vs CM; # p < 0.05 vs CF. Cm: control males; ERAm: ERA-treated males; Cf: control females; ERAf: ERA-treated females. CA: cortical area; JA: juxtamedullary area.

### Renal TBARS production

**Fig 3:**
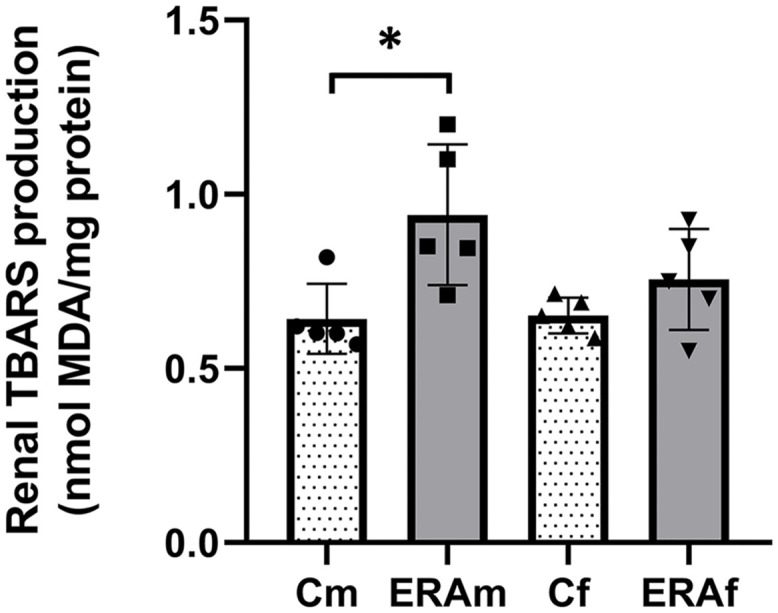
Renal TBARS production in 7-day-old rats. Thiobarbituric acid-reacting substances (TBARS) production in renal homogenates of 7-day-old rats. Data are expressed as mean ± SEM (n = 5/experimental group). Data were analyzed using two-way ANOVA followed by the Bonferroni post-test. Treatment with ERA had a significant overall effect (p<0.005). *p < 0.05 vs indicated group. Cm: control males; ERAm: ERA-treated males; Cf: control females; ERAf: ERA-treated females.

Two-way ANOVA showed an effect of ERA treatment (p < 0.005). Despite this significant effect in ERA treatment, Bonferroni post-test showed that TBARS levels were significantly higher only in ERAm vs Cm (p < 0.05) (Fig 3).

### NO to O_2_^-^ ratio in renal mitochondria

**Fig 4:**
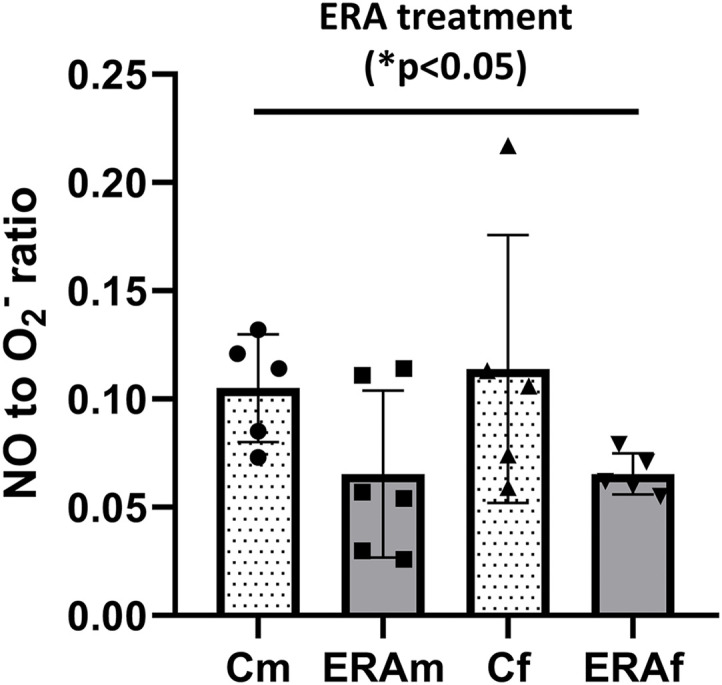
Nitric oxide (NO) to Superoxide anion (O_2_^-^) ratio in renal isolated mitochondria of 7-day-old rats. Data are expressed as mean ± SEM (n = 5/experimental group). Data were analyzed using two-way ANOVA followed by the Bonferroni post-test. Treatment with ERA had a significant overall effect (p < 0.05). Cm: control males; ERAm: ERA-treated males; Cf: control females; ERAf: ERA-treated females.

ERA treatment had an overall effect decreasing the NO to O_2_^-^ ratio in renal mitochondria (p < 0.05). (Fig 4).

The underlying data to generate the ratio NO/O^-^_2_ are included in Supplementary fig 1 (S1 Fig). S1 Fig shows that there were no significant changes in mitochondrial NO production between groups. However, sex and treatment had an overall effect on O_2_^-^ mitochondrial production (p < 0.05), showing the highest levels of O_2_^-^ production in ERAm.

### Nos1 and Nos3 mRNA expression levels in renal homogenates

**Fig 5:**
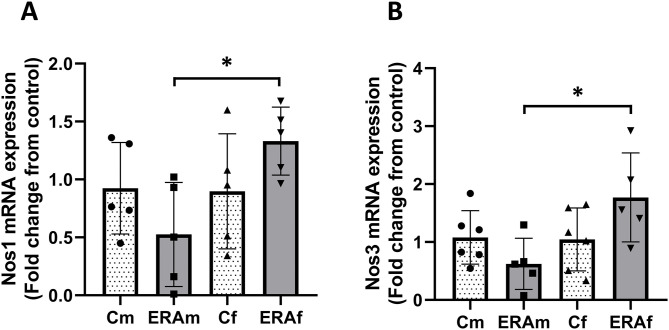
*Nos1* and *Nos3* mRNA expression levels in renal homogenates of 7-day-old rats. (A) *Nos1* mRNA expression levels (B) *Nos3* mRNA expression levels. mRNA levels are expressed as relative values from control rats. Data are expressed as mean ± SEM of at least five independent experiments. Data were analyzed using two-way ANOVA followed by the Bonferroni post-test. There was a statistically significant interaction between the effects of ERA treatment and sex on *Nos1* and *Nos3* mRNA expression levels (p < 0.05). *p < 0.05 vs. indicated groups. Cm: control males; ERAm: ERA-treated males; Cf: control females; ERAf: ERA-treated females.

A significant interaction between sex and ERA treatment was observed (p < 0.05) for both *Nos1* and *Nos3* mRNA expression levels, indicating that ERA treatment effect on these variables is sex-dependent. Bonferroni post-test analysis revealed that ERAm had reduced Nos1 and Nos3 mRNA expression levels compared to ERAf (Fig 5).

### NADPH-diaphorase activity in renal cortical structures

Table 4 shows NADPH-d activity (expressed as arbitrary units of OD) in different cortical renal structures. A significant interaction between sex and ERA treatment was detected for NADPH-d activity in immature structures. Bonferroni post-test revealed that ERAm exhibited a reduced NADPH-d activity in immature structures compared to Cm. In addition, ERA treatment exerted a significant overall effect on the macula densa (p < 0.01). No changes in NADPH-d activity were found in glomeruli or proximal tubules between experimental groups.

**Table 4:** NADPH-diaphorase activity in renal cortical structures of 7-day-old rats.

	Cm	ERAm	Cf	ERAf
Glomeruli	0.598 ± 0.023	0.535 ± 0.028	0.562 ± 0.028	0.526 ± 0.025
Immature structures	0.664 ± 0.041	0.557 ± 0.011*	0.595 ± 0.019	0.597 ± 0.013
Proximal tubules	0.633 ± 0.015	0.673 ± 0.024	0.623 ± 0.045	0.589 ± 0.021
Macula densa	0.700 ± 0.026	0.615 ± 0.005	0.746 ± 0.042	0.674 ± 0.009

The values are expressed as optical density (OD arbitrary units) (mean ± SEM; n = 5/experimental group). Data were analyzed using two-way ANOVA followed by the Bonferroni post-test. There was a significant interaction between the administration of ERA and sex on immature structures (p < 0.05). Treatment with ERA had a significant overall effect on the macula densa. *p < 0.05 vs Cm. Cm: control males; ERAm: ERA-treated males; Cf: control females; ERAf: ERA-treated females.

**Fig 6:**
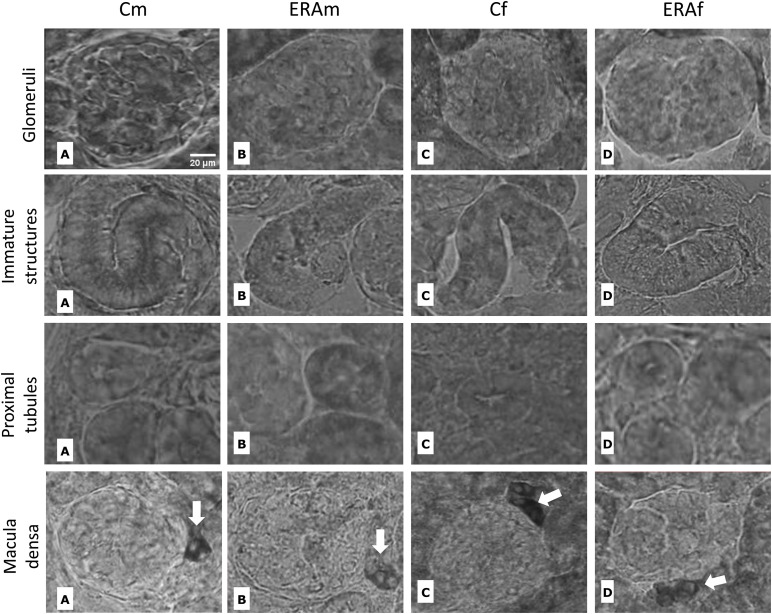
NADPH-diaphorase activity in renal cortical structures of 7-day-old rats. Representative images of NADPH-diaphorase activity assay in different renal structures. A = control males; B = ERA-treated males; C = control females; D = ERA-treated females. White arrows show macula densa cells. Total magnification: 400x.

Representative images of the different structures analyzed are shown in Fig 6.

### Hydrogen peroxide production and antioxidant enzyme activity in renal mitochondria

**Fig 7:**
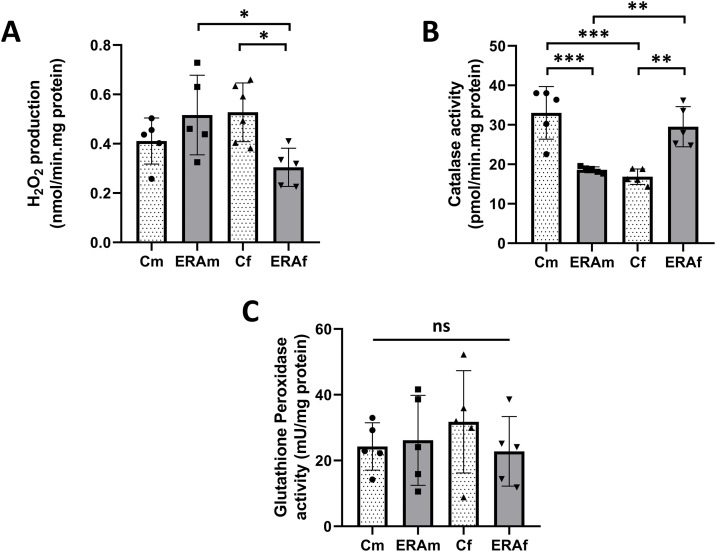
Hydrogen peroxide production rate and antioxidant enzyme activity in renal mitochondria of 7-day-old rats. (A) Hydrogen peroxide (H_2_O_2_) production rate in freshly isolated renal mitochondria. (B) Catalase activity in renal mitochondria. (C) Glutathione Peroxidase activity in renal mitochondria. Data are expressed as mean ± SEM of at least five independent experiments. Two-way ANOVA showed a statistically significant interaction between the effects of ERA treatment and sex on Catalase activity (p < 0.0001) and H_2_O_2_ production (p < 0.01). *p < 0.05, **p < 0.01, ***p < 0.001 vs indicated group. Cm: control males; ERAm: ERA-trated males; Cf: control females; ERAf: ERA-treated females.

Two-way ANOVA showed a statistically significant interaction between the effects of ERA treatment and sex on H_2_O_2_ production (p < 0.01) (Fig 7A). Bonferroni post-test analysis revealed that ERAm had an increased H_2_O_2_ production compared to ERAf (p < 0.05). In addition, ERAf showed a decreased H_2_O_2_ production versus Cf (p < 0.05).

A statistically significant interaction between the effects of ERA treatment and sex was also observed on catalase activity (p < 0.0001). Bonferroni post-test analysis showed that ERA treatment decreased catalase activity in males (p < 0.01) and, conversely, increased it in females (p < 0.01) versus their respective controls. In addition, catalase activity was higher in ERAf than in ERAm (p < 0.05) (Fig 7B).

No significant changes were found in GPx activity between experimental groups (Fig 7C).

## Discussion

The “Developmental Origins of Health and Disease” (DOHaD) theory postulates that some adult diseases, such as hypertension and kidney disease, originate in early life. The exposure to adverse stressors during development induces the adaptation of the genetic program, leading to structural, physiological, and metabolic changes in different organs, predisposing to diseases in adult life [29]. Given the kidney´s vulnerability to early-life stressors, renal programming is key in the developmental programming of hypertension and renal diseases [29,30]. In rats, nephrogenesis begins on embryonic day 12, but renal development continues during the early postnatal period [31]. Thus, both the fetus and the neonate are at risk of renal programming [32].

Previously, we demonstrated that the administration of a dual ERA during the first 21 days of life of SD rats decreases glomerular number in the renal juxtamedullary area in both ERAm and ERAf by histomorphometry and only in ERAm by the acid maceration method. The decreased glomerular number could be due to decreased cellular proliferation and/or increased apoptosis [1]. In the current study, we found no changes in renal cellular proliferation, but found an increased number of apoptotic nuclei in 7-day-old ERAm and ERAf, specifically in the juxtamedullary area, consistent with our previous work [1].

These results align with those described by Yoo et al., who showed that ERA administration from PD 1–7 increases apoptotic cells in the developing rat kidney, but, contrary to our results, they found a reduced number of PCNA-positive renal cells [8]. The differences with our results could be due to the type of ERA treatment: while these authors administered an ERA that specifically blocked ETA receptors, we used an ERA that blocks both ETA and ETB receptors. Another difference with our study is that Yoo et al. showed a reduced kidney size after ETA blockade, while the treatment with a dual ERA did not affect body weight, kidney weight or kidney weight/100 g b.w. in 7-day-old male and female rats, despite the increased apoptosis observed in the juxtamedullary area.

A mechanism for increased apoptosis is oxidative stress [14]. During development, cell metabolism increases, producing large amounts of reactive oxygen and nitrogen species (ROS/RNS). To ensure proper development, cells must protect themselves from the potential deleterious effects of these species [33]. However, when the capacity of the antioxidant system is surpassed, ROS can induce tissue damage and impaired organ functionality [33]. In this study, ERAm showed increased renal TBARS levels when compared with Cm, suggesting increased renal lipid peroxidation.

Additionally, the NO pathway was impaired, with a decreased NADPH-d activity in immature structures that originate the glomeruli in ERAm vs Cm, but not in ERAf. Besides, we found an overall significant effect of ERA treatment with lower values of NADPH-d activity in the macula densa of ERA-treated rats vs controls. The effects of ERA treatment on *Nos1* and *Nos3* mRNA renal expression were sex-dependent, decreasing *Nos1* and *Nos3* mRNA renal expression levels in males and increasing their expression in females, suggesting that ERAf are more protected than ERAm.

An imbalance between NO and ROS under oxidative stress favors oxidative reactions in organs involved in the control of blood pressure, such as the kidneys, resulting in the development of hypertension [34]. Here, we found that ERA treatment had an overall effect, decreasing NO to O_2_^-^ ratio in ERA-treated rats vs controls. Interestingly, Tain et al. reported that asymmetric dimethylarginine, a ROS inducer and endogenous NOS inhibitor, inhibited ureteric bud branching morphogenesis, leading to decreases in nephron number [16]. Considering these findings, the impaired NO pathway and the decreased NO to O_2_^-^ ratio seen in 7-day-old ERA-treated rats could be linked to the lower glomerular endowment that we had found previously in ERA-treated 21-day-old rats [1].

On the other hand, NO and O_2_^-^ exert opposing effects on both vascular and tubular function at the renal level, and the balance between them modulates renal sodium handling. NO promotes water and sodium excretion, favoring BP reduction, while O_2_^-^ promotes water and salt retention, leading to a BP increase [35]. Imbalance of the NO to O_2_^-^ ratio alters renal hemodynamics and renal excretory function, leading to salt retention, contributing to the development of salt-sensitive hypertension [36].

Another important non-radical ROS is H_2_O_2_. ERA treatment effect on H_2_O_2_ renal mitochondrial production was sex-dependent: ERA treatment increased H_2_O_2_ production in males, while decreased it in females. ERA treatment also influenced catalase activity in a sex-dependent manner. ERAm showed decreased catalase activity, while ERAf showed increased catalase activity, suggesting that females treated with a dual ERA during the early postnatal period may be more protected than males. The increased H_2_O_2_ production in ERAm may contribute to hydroxyl radical formation and could explain the increase observed in renal TBARS levels [37]. We found no changes in the activity of GPx, another important antioxidant enzyme, between experimental groups.

Previous research articles studying renal oxidative stress and its relationship with hypertension of developmental origins in animal models focus mainly on the NO pathway and oxidative stress markers, such as 8-hydroxydeoxyguanosine, and evaluate the offspring after weaning or during adulthood [12,38,39]. In this work, we investigated not only the renal NO pathway and TBARS levels, but also antioxidant enzymes such as catalase and GPx, and renal ROS, such as H_2_O_2_, in renal tissue during the early postnatal period.

Oxidative stress could affect not only prime organs involved in the regulation of blood pressure, but also coordinated regulatory systems such as the renin-angiotensin system and ET system [38]. Further studies are needed to elucidate whether the inhibition of the ET system during the early postnatal period could also impair kidney regulatory systems, and to establish potential links between the altered regulatory systems and renal programming in this experimental model.

Previously, we reported that males treated with a dual ERA during the early postnatal period increased their systolic blood pressure after a high-sodium diet in adulthood, showing impaired capacity to excrete sodium and water with an increased renomedullary expression of aquaporin-2 and α-epithelial sodium channel and a decreased renomedullary ET-1 production [2]. Thus, the early imbalance between NO and O_2_ favoring O_2_^-^ over NO observed in ERA-treated rats could be one of the mechanisms driving salt sensitivity.

Finally, several studies have established a link between ROS generation and epigenetic marks: the increase in ROS levels may be one of the downstream mediators that initiates epigenetic changes and renal programming in offspring [40]. Considering these findings, the early-life renal oxidative stress observed in ERA-treated rats may induce epigenetic modifications contributing to salt sensitivity in adulthood. These modifications may lead to altered renal expression of proteins involved in sodium and water handling or to altered expression of proteins that regulate blood pressure. Further research is required to determine whether ERA-treated rats exhibit distinct epigenetic marks compared to controls and to establish a potential link between these marks and early-life renal oxidative stress.

In summary, this study shows that the alterations previously observed in the kidneys of ERA-treated rats during the postnatal period (decreased glomerular number, decreased juxtamedullary filtration surface area and glomerular filtration rate) could be due to an early imbalance between NO and ROS, increasing renal oxidative stress and leading to a misbalance between proliferation and apoptosis increasing apoptosis at the level of the juxtamedullary cortex, especially in the structures that originate the glomeruli. We also showed some sex differences, especially in *Nos1* and *Nos3* expression, in H_2_O_2_ production and in catalase activity, suggesting that females are more protected than males, even at an early stage of development, without the influence of sex hormones.

Understanding the molecular mechanisms underlying endothelin inhibition in the early postnatal period could have potential utility for the design of reprogramming strategies.

## Supporting information

S1 Fig NO and O_2_^-^ production in renal mitochondria. Nitric oxide (NO) and Superoxide anion (O_2_^-^) production in renal isolated mitochondria of 7-day-old rats. Data are expressed as mean ± SEM (n = 5/experimental group). Data were analyzed using two-way ANOVA followed by the Bonferroni post-test. Sex and ERA treatment had a significant overall effect on O_2_^-^ production (*p < 0.05); ns = non-significant. Cm: control males; ERAm: ERA-treated males; Cf: control females; ERAf: ERA-treated females.
(TIF)
